# Severe lumbar radiculopathy with epidural venous plexus engorgement in a morbidly obese pediatric patient: A case report

**DOI:** 10.1097/MD.0000000000016842

**Published:** 2019-08-16

**Authors:** Hee Joon Jeong, Woo Seog Sim, Hue Jung Park, Seung Hwan Lee, Min Seok Oh, Min Kyoung Cho, Heui Jin Seon, Jin Young Lee

**Affiliations:** a Department of Anesthesiology and Pain Medicine, Samsung Medical Center, School of Medicine, Sungkyunkwan University; b Department of Anesthesiology and Pain Medicine, Seoul St. Mary's Hospital, College of Medicine, The Catholic University of Korea, Seoul, Korea.

**Keywords:** engorgement, epidural venous plexus, lumbar radiculopathy

## Abstract

**Introduction:**

Engorgement of the epidural venous plexus (EVP) is a rare cause of nerve root impingement. Dilated epidural veins cause compression of the thecal sac and spinal nerve roots, leading to lumbar radiculopathy.

**Patient concerns:**

Here we describe a case of severe lumbar radiculopathy in a 15-year-old morbidly obese boy.

**Diagnosis:**

Enhanced lumbar magnetic resonance imaging revealed left sided L1–L2 disc protrusion and engorgement of the lumbar EVP, resulting in narrowing of the thecal sac in the entire lumbar spine. There was no evidence of an intra-abdominal mass, thrombosis of the inferior vena cava, or vascular malformation.

**Interventions:**

A caudal epidural block was administered under fluoroscopic guidance. The patient reported a 30% reduction in pain intensity for just 1 day.

**Outcomes:**

The patient has been followed up for 2 years. He continues to take medication, including morphine sulfate 15 mg, gabapentin 300 mg, and oxycodone 20 mg per day. He is on a diet with exercise for weight reduction.

**Conclusion:**

An engorged EVP should be considered in the differential diagnosis of radiculopathy in morbidly obese patients.

## 1 Introduction

Back pain and radiculopathy are commonly caused by disc herniation or spinal stenosis. These pathologies can cause compression, inflammatory changes, and ischemic injury of the involved neural structures.^[1]^ Engorgement of the epidural venous plexus (EVP) has rarely been reported as a cause of radiculopathy in the literature.^[2,3,4]^ Here we describe the case of a morbidly obese patient with severe lumbar radiculopathy who had thecal sac narrowing as a result of EVP engorgement. This case emphasizes the importance of careful history-taking, clinical examination, and neuroimaging when diagnosing the cause of radiculopathy in a morbidly obese patient.

## 2 Case report

**Figure 1:**
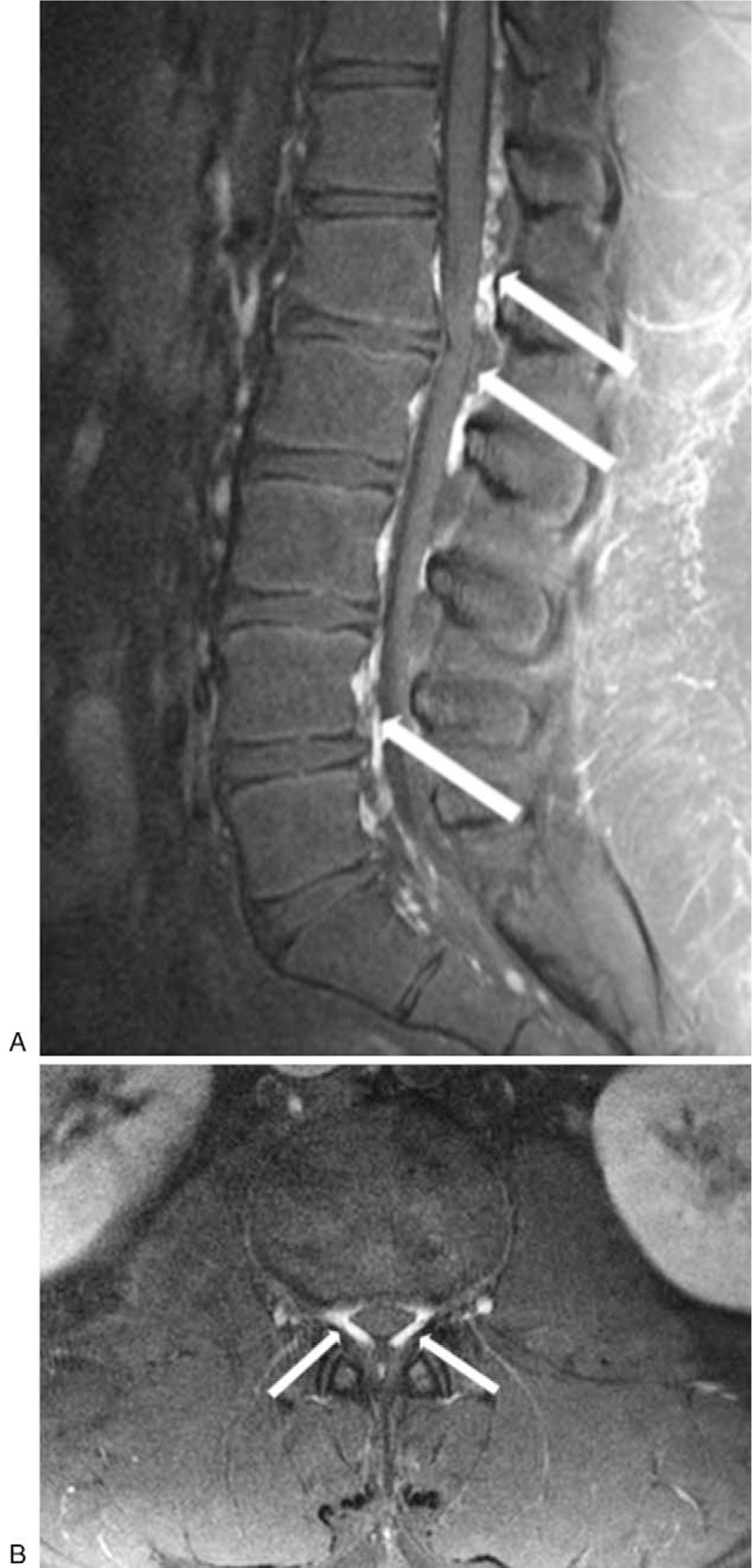
T2-weighted sagittal (A) and axial (B) magnetic resonance images show enlarged lumbar epidural veins (arrow) compressing the dural sac and causing disc herniation at the left L1–L2 level.

This research was approved by our departmental ethics committee (SMC 2019–01–019). A database from a university pain clinic was retrospectively reviewed for the period from May 2016 to August 2018. Informed written consent was obtained from his parents for publication of this case report and accompanying images. A 15-year-old boy (height, 180 cm; body weight, 128 kg; body mass index, 39.5) presented to our pain clinic with complaints of lower back pain and left leg pain. Two years earlier, he had been diagnosed with left-sided renal stones. He had undergone retrograde intrarenal surgery and insertion of a ureteric stent 4 months previously. Although most of the stones had been removed, his lower back pain and left leg pain worsened, with an intensity of 7 to 9 on a visual analog scale (VAS, 0 = no pain; 10 = worst pain imaginable). His pain was partially relieved (VAS 4–6) by morphine sulfate 15 mg daily. He was referred to our pain clinic because of the persistent pain and concerns about chronic use of opioids. On initial examination, he had continuous throbbing pain and radicular numbness in his left leg. He could not stand for more than 10 minutes because of the pain. Neurological examination revealed decreased sensation of 4/5 (0 = no sensation; 5 = normal sensation) in the distribution of the left L1–L3 dermatomes. Motor strength in left hip flexion and left knee extension was graded 4/5. The preliminary diagnosis was disc herniation. Enhanced lumbar magnetic resonance imaging (MRI) revealed left sided L1–L2 disc protrusion and engorgement of the lumbar EVP, resulting in narrowing of the thecal sac in the entire lumbar spine (Fig. 1). Abdominal computed tomography was performed to exclude the possibility of an abnormality in the venous circulation. There was no evidence of an intra-abdominal mass, thrombosis of the inferior vena cava, or vascular malformation. Liver biopsy revealed moderate steatohepatitis. A caudal epidural block was administered under fluoroscopic guidance for pain management. Five milliliters of 0.75% ropivacaine, dexamethasone 5 mg, hyaluronidase 750 IU, and normal saline were infused into the epidural space. There were no complications and the patient reported a 30% reduction in pain intensity for just 1 day. We did not attempt further epidural interventions because of the risk of inadvertent EVP puncture and bleeding. He was followed up for 2 years, during which time the VAS score was consistently 4 to 6. He continues to take medication, including morphine sulfate 15 mg, gabapentin 300 mg, and oxycodone 20 mg per day. He is on a diet with exercise for weight reduction.

## 3 Discussion

Spinal radiculopathy is a multifactorial disease with inflammatory, neuropathic, ischemic, and mechanical components.^[1,5]^ Mechanical stress on the nerve root can impair intra-radicular blood flow and cause intra-neural damage and functional changes.^[1]^ However, vascular abnormalities causing compression and excessive pressure on a nerve root and/or dorsal root ganglion have occasionally been reported.^[6,7,8]^ These abnormalities may give rise to symptoms similar to lumbar disc herniation or spinal stenosis. The exact diagnosis can often be delayed. The reported incidence of lumbar epidural varices ranges from 0.07% to 1.2%.^[8]^ The causes of spinal radiculopathy include vascular malformation, thrombosis of the iliac vein, superior vena cava, or inferior vena cava, Budd–Chiari syndrome, intracranial hypotension, pregnancy, and portal hypertension.^[2,3,4,6]^ The venous system of the spine is a valveless network that is connected to the vena cava, pelvic veins, and azygos system.^[9]^ It communicates with multiple intersegmental pedicular branches adjacent to the spinal nerve root.^[2,10]^ The EVP distends and becomes engorged in the presence of conditions that disturb the venous drainage to the vena cava.^[2,3,7]^ The differential diagnosis should include disc herniation, spinal stenosis, tumor, abscess, hematoma, and/or perineural cyst.^[4,9,11]^ Treatments include improving venous drainage, antithrombotic medication, and removal of thrombus or varicosity by surgery. Kawai et al^[7]^ have reported a case of radicular pain caused by compression of the lumbar nerve roots by enlarged veins because of idiopathic epidural lipomatosis in a nonobese patient. Cardiopulmonary dysfunction causing cyclic distension of the lumbar EVP has been reported.^[4,10]^ The influence of obesity on EVP engorgement remains unclear. A previous study demonstrated that if the inferior vena cava is occluded by increased abdominal pressure because of the weight of the abdominal contents in obesity or increased epidural fat, blood flow through the lumbar vertebral plexus can increase and the extradural vein will distend.^[12]^ In contrast, another study showed that the amount of epidural fat does not appear to be correlated with body mass index.^[13]^ Even though we could not test the relationship between obesity and EVP engorgement in our case, we suspect that obesity might have caused increased abdominal pressure and a compressive force on the EVP, leading to compression of the thecal sac and lumbar radiculopathy in addition to left sided L1–L2 disc herniation. Follow-up lumbar MRI after significant weight reduction would be needed to evaluate this possibility. However, our patient refused to undergo follow-up MRI, which limited our ability to confirm the correlation of obesity with an engorged EVP. Obesity has been reported to be a risk factor for neuropathy and might have aggravated this patient's radicular pain.^[14]^

We have reported a case of severe lumbar radiculopathy related to EVP engorgement that might have been caused by increased abdominal pressure as a result of morbid obesity. Without MRI, this type of pathology would be indistinguishable from other pathologies. Therefore, an engorged EVP should be considered in the differential diagnosis of radiculopathy in morbidly obese patients.

## Author contributions

**Conceptualization:** Woo Seog Sim, Hue Jung Park, Jin Young Lee.

**Data curation:** Hee Joon Jeong, Min Seok Oh, Min Kyoung Cho, Jin Young Lee.

**Investigation:** Hee Joon Jeong, Jin Young Lee.

**Supervision:** Jin Young Lee.

**Validation:** Min Seok Oh, Min Kyoung Cho.

**Visualization:** Seung Hwan Lee, Heui Jin Seon.

**Writing – original draft:** Hee Joon Jeong, Jin Young Lee.

**Writing – review & editing:** Jin Young Lee.

Jin Young Lee orcid: 0000-0003-1499-2197.
